# Organoids: An Emerging Precision Medicine Model for Prostate Cancer Research

**DOI:** 10.3390/ijms25021093

**Published:** 2024-01-16

**Authors:** Mohammad Waseem, Bi-Dar Wang

**Affiliations:** 1 Department of Pharmaceutical Sciences, School of Pharmacy and Health Professions, University of Maryland Eastern Shore, Princess Anne, MD 21853, USA; mwaseem@umes.edu; 2 Hormone Related Cancers Program, University of Maryland Greenebaum Comprehensive Cancer Center, Baltimore, MD 21201, USA

**Keywords:** prostate cancer, organoids, 3D model, precision medicine, in vitro and in vivo models, castration resistant prostate cancer (CRPC), neuroendocrine prostate cancer (NEPC)

## Abstract

Prostate cancer (PCa) has been known as the most prevalent cancer disease and the second leading cause of cancer mortality in men almost all over the globe. There is an urgent need for establishment of PCa models that can recapitulate the progress of genomic landscapes and molecular alterations during development and progression of this disease. Notably, several organoid models have been developed for assessing the complex interaction between PCa and its surrounding microenvironment. In recent years, PCa organoids have been emerged as powerful in vitro 3D model systems that recapitulate the molecular features (such as genomic/epigenomic changes and tumor microenvironment) of PCa metastatic tumors. In addition, application of organoid technology in mechanistic studies (i.e., for understanding cellular/subcellular and molecular alterations) and translational medicine has been recognized as a promising approach for facilitating the development of potential biomarkers and novel therapeutic strategies. In this review, we summarize the application of PCa organoids in the high-throughput screening and establishment of relevant xenografts for developing novel therapeutics for metastatic, castration resistant, and neuroendocrine PCa. These organoid-based studies are expected to expand our knowledge from basic research to clinical applications for PCa diseases. Furthermore, we also highlight the optimization of PCa cultures and establishment of promising 3D organoid models for in vitro and in vivo investigations, ultimately facilitating mechanistic studies and development of novel clinical diagnosis/prognosis and therapies for PCa.

## 1. Introduction

According to the USA demographic investigations, about 20 million new cases and 10 million deaths from cancer are anticipated every year. The global burden has a prediction of about 30 million new cancer cases by 2040, indicating an expanding incidence of cancer in the upcoming years. In the United States, about 2 million (1,958,310) new cancer cases were reported in 2023. Among the 2 million new cases, cancer prevalence in men was documented as about approximately 1 million (1,010,310). Currently, in males, the most prevalent cancers occurred in genital organs (299,540), prostate (288,300), and digestive organs (194,980). Notably, prostate cancer (PCa) is the most frequently diagnosed cancer (288,300 estimated new cases in 2023) and the second leading cause of cancer deaths (34,700 estimated deaths in 2023) among men in the USA [1].

In PCa, androgen receptor signaling plays a critical role in tumor growth and survival. Androgen deprivation therapy (ADT), therefore, has been established as one of the major standards for PCa care [2]. However, the tumors in some ADT-treated patients eventually progressed to a more aggressive form, called castration-resistant PCa (CRPC). CRPC exhibits a significant increase in prostate-specific antigen (PSA) value and/or radiographic progression despite castration [3,4]. Currently, the approved therapies for CRPC include chemotherapy, immunotherapy, radiation therapy, targeted therapies and hormone therapies (i.e., by AR inhibitors) [5]. Although several novel therapeutic attempts have been developed, metastatic CRPC still remains a lethal disease with a survival rate at <2–3 years from the time of progression [6].

**Figure 1:**
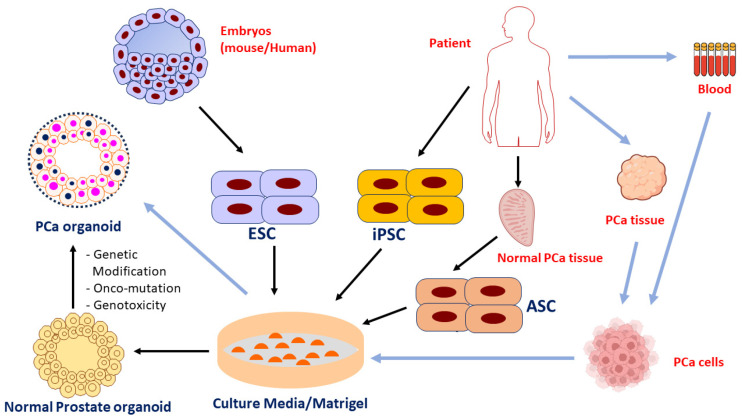
Development of PCa organoids. (1) PCa organoids can be generated from tissues and blood samples of PCa patients. The PCa tissues and blood samples cultured in Matrigel-containing medium are eventually formed 3D PCa organoids (procedures are indicated by the blue arrows). (2) Organoids can be developed from embryonic stem cells (ESCs), induced pluripotent stem cells (iPSCs), and patient-derived adult stem cells from both normal and cancerous tissues (ASCs). These ESC and iPSC cells can be cultured in Matrigel-containing medium and formed normal prostate organoids. After multiple genetic mutations/modifications, onco-mutations, and/or exposures to genotoxicity, these non-tumor organoids have the opportunity to be developed into PCa organoids (procedure indicated by solid black arrows).

Considerable progress has been observed in PCa therapeutic strategies; however, a more effective treatment still remains to be developed considering the tumor heterogeneity in patients. Therefore, there is an urgent need for developing a high-fidelity preclinical model that provides a precise insight into PCa-related molecular signature and genetic alterations for conducting basic research and to translate the results into clinical applications for personalized PCa detection and therapy. One of the most promising strategies has been the development of organoids as novel in vitro or ex vivo model systems for both basic and translational PCa researches [7]. Organoids have been considered as the cultures of epithelial cells that are capable of proliferating as multicellular structures at semisolid matrix platform in the presence of mitogens and other pathway modulators. An organoid is defined as a model of “resembling tissue or organ” and considered as an in vitro 3D culture system that can be derived from self-organizing stem cells. In addition, organoids mimic the in vivo structures and functions of the organ. Organoids can also be derived from either pluripotent stem cells (PSCs) or organ-specific adult stem cells (ASCs). PSCs, for instance, can be used to generate in vitro non-cancerous organoid models recapitulating the overall molecular process of organogenesis, where tissues are derived from embryonic stem cells (ESCs). Meanwhile, tissue and blood samples can be further cultured in a Matrigel-containing medium to develop 3D PCa organoids (Figure 1). Organoids are ideal model systems for characterization of both cellular and molecular events in PCa, and have the advantage to be genetically manipulated via molecular approaches to pinpoint the mechanisms underlying cancer etiology and cancer drug resistance [8]. In recent years, three-dimensional (3D) organoid cultures of human tumor cells and/or tissues have evolved as a relatively low-cost and representative platform for a 3D cancer model recapitulating its heterogeneity nature and interactions with the tumor microenvironment under in vitro state [9]. In this review, we discuss PCa-based organoids as 3D models generated from neoplastic cells and/or patient specimens that mimic the key alterations at genetic, histopathological, and phenotypic levels of parent cancer cells. We also discuss the potential of utilizing PCa organoids as models to study PCa microenvironment, identify critical genetic mutations/alterations, and assess drug sensitivity/resistance. Furthermore, we summarize the promising potentials of organoids in clinical applications, including development of precision diagnosis/prognosis biomarkers, novel therapeutics, and personalized treatment for PCa diseases.

## 2. Background and Recent Stage of Prostate Organoid Culture

**Figure 2:**
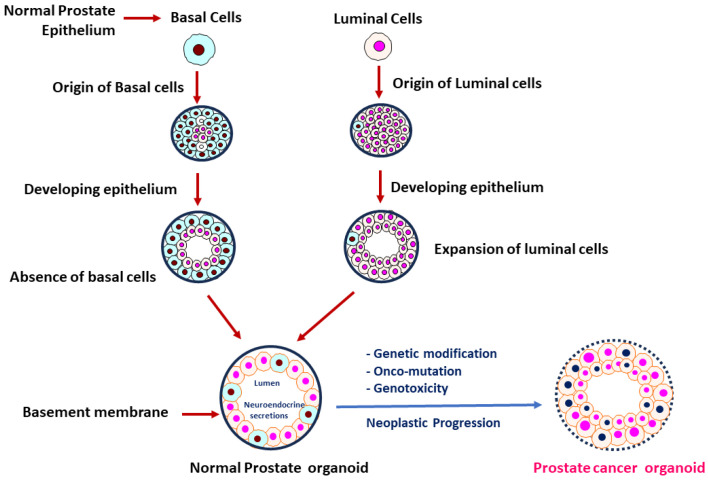
The majority of PCas are defined by abnormal luminal cell development and lack of basal cells when compared to prostate tissues with normal basal and luminal cells. During transformation to neoplasm, impairment of prostatic epithelium was characterized. The normal prostatic cluster of cells (normal prostate organoid, **Left**) consists of an epithelial compartment comprising of basal (sky blue) and luminal (light orange) cells, and a limited population of neuroendocrine cells that act as stem cells in response to repairing the cellular damage. During neoplastic progression, a normal prostate organoid is transformed into a PCa organoid through multiple genetic alterations and onco-mutations. We note that a PCa organoid (**Right**) is characterized by luminal hyperproliferation, breakdown of basement membrane, loss of basal cells, prominent nucleoli (blue/pink), and nuclear enlargement.

The earlier development of prostate 3D culture technology was achieved by establishing mouse-derived spheroid cultures. Such methodology was performed by culturing whole or fractionated mouse prostate epithelium in commercially available serum-free medium coupled with a 3D extracellular matrix (ECM), finally facilitating the development of spheroids with self-renewing and self-organizing differentiation potentials [10,11]. The usage of Matrigel in the development of organoid culture has been pinpointed as a key component for early success for developing organoids. These early strategies allowed scientists to develop 3D prostate cultures by continuing the propagation of luminal cells followed by the addition of androgens such as dihydrotestosterone (DHT) to the medium. This approach resulted in a limited luminal differentiation to intermediate phenotypes with AR expression; however, the secretory phenotype of prostate luminal epithelium was still missing in the established spheroid models [12]. Later, the 3D normal prostate organoids consisting of both basal and luminal cells were developed [11,13], and 3D PCa organoids were developed from metastatic PCa biopsies and circulating PCa cells [11]. These 3D models (Figure 2) of normal prostate organoids and PCa organoids (developed from PCa tissues/circulating cells, or through neoplastic transformation of normal prostate organoids) can be utilized to address the key molecular mechanisms underlying PCa development and/or progression.

In recent years, Julio and her colleagues developed a novel strategy for generation of PCa organoids to evaluate therapeutic responses [14]. This group optimized the protocol for efficiently generating patient-derived organoids (PDOs), and detailed characterization of PCa organoids was provided. Furthermore, in collaboration with the NEXUS Personalized Health Technologies, Julio et al. further developed a PDO for medium-throughput screening for drug efficacies. In this study, the authors demonstrated that PDOs maintain the molecular and cellular features of the original prostate carcinoma that the PDOs were derived from. For instance, the PDOs share comparable genomic landscapes and gene expression profiles when compared to parental prostate carcinoma [14].

In 2009, Sato et al. [15] defined the organoid culture medium by including stem cell niche components, including Wnt pathway agonist R-spondin-1, epidermal growth factor (EGF), and the bone morphogenetic protein (BMP) antagonist Noggin. This formulation efficiently improved the long-term self-renewal and differentiation potential of mouse intestinal crypt stem cells into a 3D ECM. Using basal medium containing nicotinamide, prostaglandin E2 (PGE2), and components that inhibit TGF-β and MAPK signaling, Jung et al. successfully established human intestinal organoids. These intestinal organoids were developed from cocultures containing intact intestinal crypts and isolated stem cells derived from normal or tumor tissues [16]. Employing various stem cell-promoting culture conditions, several epithelial tumor organoids were established. For example, after optimization of tissue-specific factors (such as adding estrogen or testosterone), subtypes of breast cancer organoids or PCa organoids were established [17].

Earlier studies conducted by Clevers and colleagues demonstrated a PCa organoid model that was developed through continuous growth and differentiation of prostate luminal epithelial stem cells. This organoid model has been used to study the role of R-spondin, Noggin, and testosterone [8,18]. Prostate-specific modification of intestinal medium and other changes in 3D culture conditions [19] have led to a robust procedure for developing mouse-derived prostate organoids [20].

Gao and colleagues reported the development of PCa organoid culture from metastatic CRPC patient tissues [17]. However, various success rates of developing sustainable CRPC organoids from patient tumor needle biopsies have been reported. An average of ≤10% success rates of developing CRPC organoids are observed, which is significantly lower than the rates for many other epithelial tumor organoids (such as pancreatic, colorectal, and breast cancer organoids). These results reflect the complexity of tumor heterogeneity of CRPC and also suggest a need to further optimize the culture conditions for establishing fidelity CRPC organoid models [8].

In recent years, an increasing number of new PCa organoid models have been established from metastatic PCa tissue biopsies. These new models are highlighted to represent the most important PCa subtypes, such as AR-driven (AR^+^) adenocarcinoma, AR-independent neuroendocrine positive (AR^−^ NE^+^), or double-negative (AR^−^ NE^−^) PCa [17,21,22,23]. In contrast, an organoid model derived from castration-sensitive PCa has also been established [14]. Furthermore, modified culture conditions have been applied to develop PCa organoids from patient-derived xenograft (PDX), aiming to establish novel PCa organoid models mimicking the PCa heterogeneity [21].

## 3. Tumor Model System in PCa

PCa represents the most common cancer type in males, and it has gained an increasing attention all over the world. In male population, it has also been ranked among the highest level of cancer-associated mortality [24]. Several studies have utilized organoids as ex vivo models to evaluate tumor microenvironment (TME) and translational medicine in tumor biology [11,19,22,25,26,27,28,29,30,31,32]. Heninger et al. established patient-derived organoids (PDOs) from locally advanced PCa [33]. According to their orthogonal analyses, these organoids reserved and maintained the complexity of TME as observed in parental PCa. According to orthogonal flow cytometry analysis, PCa organoids retained a distinct subpopulation of epithelial cells and reserved the expression signature of AR and AR-related genes as parental PCa cells [33]. Using mouse prostate organoids, Grbesa et al. revealed that mutations in *SPOP* (the most frequently mutated tumor suppressor gene in human primary PCa) contribute to AR accessibility and binding patterns that are similar to those in primary PCa [34].

Gao and his group developed an optimized condition wherein the continuous propagation of normal basal and luminal prostate epithelial cells was considered in the development of PCa organoid models. In this study, PCa organoids were derived from different PCa sources, including cell lines (DU-145, PC-3, LAPC4, 22Rv1, LNCaP, and VCaP), PCa tissues, iPSCs, CDXs, PDXs and circulating tumor cells. These PCa organoids have been reported to recapitulate the different molecular characterization of PCa subpopulations from different sources. Notably, the PCa organoids derived from cell lines exhibited similar molecular signatures to both primary and metastatic PCa. For instance, mutations in *SPOP*, loss/deletion of *PTEN*, *TMPRSS2-ERG* expression, or mutations in *TP53*, *PIK3R1* and/or *FOXO1* (frequently occurred in CRPCs) were retained in these PCa organoid models. In addition, these organoid models represent a series of diverse subgroups of CRPCs, including AR-dependent adenocarcinoma, AR-negative adenocarcinoma, and neuroendocrine carcinoma [17].

In addition, Clevers et al. developed a mini-gut-based 3D culture procedure for the development of primary mouse and human PCa organoids consisting of differentiated basal and luminal cells [35]. Compared to other PCa organoids, luminal cell-derived organoids seem to be better resembled to mimic the prostate gland. Moreover, a previous report showed that long-term cultured organoids are genetically stable, and they can reconstitute prostate glands through tissue recombination approaches [13]. Furthermore, this organoid system, developed in Matrigel/EGF-based culture supplemented with androgens, has been reported as a feasible model system for PCa study [19].

### 3.1. In Vitro and In Vivo 3D PCa Models

Current in vitro tumor models, such as PCa cell lines, show the limitations on featuring the genetic signature and molecular alterations compared to PCa tissue samples and PCa development/progression. Instead, PCa organoids are considered as 3D models with context of tumor microenvironment and heterogeneity, thereby serving as ideal model systems for mechanistic studies and drug screening assays [36]. Several 3D models were introduced described below.

#### 3.1.1. Spheroids

Tumor spheroids and tumorspheres are two common spherical cancer models. Formation of tumorspheres is achieved by clonal proliferation of tumor cells in low-adherent conditions with stem cell medium, while tumor spheroids are formed by aggregation and compaction of multiple tumor cells in nonadherent conditions [37]. Tumorsphere models have been used in several PCa studies. For example, co-inhibition of glucocorticoid receptor (GR) and β-catenin (by CORT-108297 and MSAB) resulted in reducing/suppressing PCa tumorsphere formation and stemness while sensitizing the resistant PCa to docetaxel [38]. In addition, PCa tumorspheres, derived from VCaP cells, were used to assess the inhibitory effects of Stattic and Napabucasin (STAT3 inhibitors) in PCa metastasis [39].

Spheroids are categorized as 3D cell models derived from cancer cell lines or patient-derived cancer samples, and they are cultured in suspension by using a scaffold and/or a hydrogel-based approach [40]. Compared to the limitations of 2D models in in vitro studies, spheroids share the advantage of mimicking several in vivo tumor phenotypes including cell–cell and cell–ECM interactions. These features allow for scientists to utilize spheroids to study cell proliferation, metabolism, hypoxia, and tumor heterogeneity in PCa. Prostate tumor-derived spheroids (also named “prostaspheres”) were utilized to examine the genetic/molecular characteristics under in vitro conditions [41]. Spheroids have been described as cost-effective 3D cell models for screening drug responses. However, spheroids have also been shown to have a lack of organized form and uniformity in culture states. In recent years, microfluidic systems have further revolutionized the process for developing PCa spheroids. However, hypoxia-induced necrosis remains a challenge for maintaining tumor spheroids. Fluidic systems (such as Microwell Flow Device, MFD) allow in-well laminar flow around the spheroids, consequently reducing necrosis and increasing integrity of cellular structures. These flow-cultured spheroids demonstrate advantages for studying hypoxia adoption, metabolism and drug efficacies in a 3D condition [42].

The process of establishing spheroids starts from long-term culturing of CRC cells and healthy intestinal stem cells in the presence of Wnt, R-spondin1, EGF, and noggin [15,35,43,44]. It is known that the organoids developed from healthy intestinal stem cells in Matrigel are able to maintain their normal genome over a period of time [45]. Medium containing Matrigel, cocktail of stem cell growth factors, TGF-β receptor inhibitor (A83-01), and p38 MAPK inhibitor (SB202190) have been used as growth media for the coculturing of healthy human intestinal cells with colon cancer cells, ultimately developing CRC organoids [43]. Similar culture protocols have been adopted to develop other types of cancer organoids, including pancreatic cancer [46] and PCa [17] organoids.

As described above, organoid models have the potential to reserve the tumor heterogeneity of parental PCa. Spheroid culture is a 3D model with a higher success rate compared to organoid culture. These spheroid models have been established by using ultra-low-attachment culture plates. Furthermore, Rho-associated protein kinase inhibitor Y-27632 has been applied to substantially support the development of spheroid culture models [47,48]. In 2018, Linxweiler et al. reported spheroids as efficient 3D models generated from radical prostatectomy specimens of PCa patients [49]. This type of 3D PCa culture has been considered as a promising model for drug development and clinical applications. In this section, we recapitulate some other PCa organoid and spheroid models derived from PCa patients.

In 2019, the Saarland University developed spheroid models from radical prostatectomy of PCa patients [49]. Specifically, more than 100 spheroid models were developed using the tissues collected from 173 PCa patients. These models were demonstrated to have higher viability after maintenance of for several months, and were well-adopted to cryopreservation procedures under in vitro conditions. Among the established spheroid models, most of the tumor spheroids expressed high levels of AR, CK8, and AMACR proteins, similar to their parental PCa cells. In summary, the advance in culture technology and tissue engineering has greatly improved the success rates for the development of patient-derived spheroid, featuring the characteristics of parental tumor phenotypes and tumor heterogeneity in PCa.

#### 3.1.2. Patient-Derived Organoids (PDOs)

In the past decade, the Memorial Sloan Kettering Cancer Center has particularly highlighted the development of “MSK-PCa models”, which are patient-derived organoid (PDO) models of PCa [17]. These PCa models were primarily established from the biopsy of PCa metastases and circulating tumor cells. In this study, the success rates of the established PDO models were ranging between 15 and 20%. These PDO models could successfully form tumors after these organoids were transplanted into SCID mice. Therefore, the MSK-PCa organoid models (PDOs) are considered ideal 3D models that recapitulate the molecular diversity of different PCa subtypes and can be applied to in vivo studies.

Likewise, Weil Cornell Medicine has developed “ORG WCM organoid models” from metastatic PCa biopsies of NEPC patients [22]. Specifically, fresh tumor tissues from 25 PCa patients at metastatic stages were employed for establishing organoid models, with a success rate of 16% (4 patients out of a total of 25). The organoids were further implemented into NOD/SCID mice to establish patient-derived organoid xenografts (PDOXs). Moreover, the PDOXs were re-passaged, sub-cultured, and employed in in vitro functional assays. The metastatic tumors collected from NEPC patients were used for the development of organoids and PDOXs. These organoids and PDOXs were found to highly express CRPC-NEPC marker genes, such as *MYCN*, *PEG10*, *SRRM4*, *EZH2*, *SOX2*, *BRN2*, and *FOXA2* and low levels of *AR* and its associated signaling genes. Additionally, these organoid models were effectively utilized as 3D models for assessing drug efficacies (i.e., efficacies of EZH2 inhibitors) in CRPC and/or NEPC.

Under the optimal culture condition, the organoids were developed from basal and luminal cells. These PCa organoids maintain multipotent progenitor cells and hold up intact AR signaling [13]. In addition, PDOs were developed from metastatic and circulating tumor cells, which were shown to maintain both histological and molecular features of patient tumors. These PDOs recapitulate the genetic features of the PCa, such as expression of the *TMPRSS2-ERG* fusion gene, mutations in *SPOP*, loss of *TP53*, *PTEN* and *CHD1* [17]. In other words, drug screening assays were conducted in PDO models for assessing the drug sensitivities in the context of patient genotypes. Taken together, these results suggest PDO as a promising 3D model for PCa research [21].

#### 3.1.3. Induced-Pluripotent Stem Cell (iPSC)-Derived Organoids

Similar to PDOs, iPSCs have also been highlighted for the establishment of human prostate organoids [50,51]. Using co-culture strategies with urogenital mesenchyme, prostate iPSC-derived organoids were developed. These 3D cultures were shown to retain the features of prostate epithelial differentiations. It was reported that iPSC-derived organoids could form glandular morphology, recapitulate prostate tissue histology and express key genes such as AR, NKX3.1 (prostate specific homeobox protein) and prostate specific antigen (PSA) [52]. iPSC-derived organoids, after transformation to PCa organoids due to multiple genetic/oncogenic alterations, have been proposed as promising 3D models for examining molecular mechanisms underlying PCa disease and evaluating drug efficacies for personalized medicine. These iPSC-derived organoids have also shed light on the development of novel models for genome editing using CRISPR/Cas9 technology. According to previous studies, iPSC-derived organoids have been successfully established as 3D models for studying glioblastoma, pancreatic and prostate cancer. Ultimately, these iPSC-derived organoids represent a promising model system mimicking patient phenotypes/characteristics in vitro, thereby leading to future discovery of new therapeutic targets in different types of cancers, including PCa [53,54,55].

Organoids derived from pluripotent stem cells (PSCs) and adult stem cells (ASCs) are considered self-organized 3D models that prominently mimic and depict both biochemical and metabolic signals in PCa. These 3D cultures have also characterized the in vivo epithelial architecture, genetics, and key functions of the organ of origin [56]. PSC-mediated organoids are developed from either induced pluripotent stem cells (iPSCs, capable to generate reprogramming of adult somatic cells) or embryonic stem cells (ESCs, pluripotent, self-renewing cells that differentiate into specialized cell types in response to developmental cues). In contrast to PSC-derived organoids that recapitulate in vivo organ development, ASC-derived organoids are utilized to model adult tissue regeneration. Despite the advantage of recapitulating organ/tissue features in iPSC/ASC-derived organoids, these organoids are not capable of completely recapitulating the in vivo microenvironment. Through numerous efforts from earlier investigations, 3D culture conditions have been optimized for more efficient establishment of organoids from both healthy and cancerous prostate tissues [13,15,17,19].

#### 3.1.4. Patient-Derived Xenograft (PDX)-Derived Organoids

PDX-derived organoids are defined as 3D organoid cultures, developed from PDXs in animal models. PDX lines from various cancers have been recently demonstrated as excellent models for cancer research due to their similar genomic and phenotypic features, and tumor heterogeneity to human cancer diseases [21,57,58]. Previous studies have also shown that differential drug responses may occur in patients with distinct genomic makeups. PDX-derived organoids represent 3D model systems carrying the genetic/genomic features in different PCa patients, therefore serving as ideal model systems for studying PCa diseases [13,17,59]. In 2018, Beshiri et al. first reported the establishment of a PDX-derived organoid from the LuCaP-PDX mouse model. These PDX-derived organoids were further used for drug screening/development purposes [18,21]. Notably, the LuCaP-PDXs have been well characterized and clinically considered as a cohort of advanced PCa PDXs. Although PDX-derived organoids share promising potentials for PCa research, it remains a challenge to establish a consistent PDX-derived organoid for in vitro applications [50,51]. For instance, low success rates of developing CRPC organoids with long-term/stable phenotypes were found. Besides the low availabilities of CRPC organoids, considerable variations were observed between different CRPC organoids [51]. To address this issue, LuCaP PDX-derived organoids were developed. These organoids exhibited exponential growth modes in long-term cultures, thereby serving as ideal PDX-derived 3D models for in vitro functional assays and drug efficacy studies for PCa diseases [11,13,17,59].

#### 3.1.5. Three-Dimensional Models for PCa Epigenomic Studies

In the past decade, next-generation sequencing has emerged as a powerful technology for understanding the PCa genome, transcriptome, and epigenome [60]. Epigenetic/epigenomic alterations (i.e., histone modification, DNA methylation, etc.) have been widely investigated during the process of PCa development and progression [61]. Aberrant DNA methylation patterns have been extensively studied in several kinds of cancers, including PCa [62,63]. Hypermethylation occurs at promoter regions of APC (encoding adenomatous polyposis coli), RASSF2 (Ras associated domain family member 2), and GSTP1 (glutathione-S transferase). DNA methylation patterns have been extensively studied in various PCa diseases, and the hypermethylation signatures in tumor suppressor genes/oncogenes were suggested as potential biomarkers for primary PCa and mCRPC [61,62,64].

Previously, Zhao et al. investigated the genome-wide DNA methylation patterns in mCRPC, and the study suggested the alteration of DNA methylation pattern begins in an early stage of PCa progression [65]. Intriguingly, PDOs derived from early and advanced PCa have sustained epigenetic features similar to the original primary and advanced tumors, respectively [22,33]. Additionally, 3D models have been demonstrated as an excellent model for epigenomic studies, such as the study of DNA methylation alterations in PCa. Lu et al. and Stepper et al. have shown that fusion of DNA methyltransferase genes (*DNMT1* and *DNMT3*) to nuclease inactivated CRISPR/Cas9 (dCas9, dead Cas9) resulted in reduced DNA methylation of the target genes, through inhibiting activities of DNMT1 and DNMT3 [66,67]. Likewise, alterations in histone modifications have also been observed during the process of PCa development and progression [68]. It has been suggested that dysregulation of histone demethylases and histone methyltransferases are critically linked to PCa aggressiveness and drug resistance [69,70]. Interestingly, the application of dCas9 fusing to genes encoding histone modification enzymes is thought to be an efficient approach to study histone modifications. Utilization of this CRISPR-dCas9-based technology (a targeted epigenome editing approach) to investigate the epigenomic alterations in 3D organoid models could provide molecular insights into the mechanisms underlying epigenetic/epigenomic alterations in PCa [71,72,73].

#### 3.1.6. Strategies for PDO Development from Tumor Immune Microenvironment (TIME)

Prostate tumors have been considered as ”immunologically cold” due to limited infiltrating cytotoxic lymphocytes and suppressed myeloid populations in prostate tumor immune microenvironment (TIME) [74,75,76]. Single-cell technology has made immunotherapy studies possible at the cellular level, particularly in a coculturing system combining PCa cells with diverse immune cells such as mononuclear phagocytes (monocytes, dendritic cells, and macrophages), T cells, NK cells, B cells and mast cells [77,78,79]. Due to the immunosuppression and immunogenic impairment in prostate TIME, blockade of immune checkpoints has been shown to be ineffective for evoking strong antitumor responses in a majority of the PCa patients [80,81]. Previous studies in mouse models have been conducted with better efficacies of the modulation of immune checkpoints in the context of TIME. Such experimental models, however, still reflected the limitations on the effects of physiological/genetic variations in complex inflammatory and immunological interactions [82]. To elucidate the complex mechanisms underlying immunotherapeutic response in PCa, there is an urgent need to establish novel 3D models with compatible prostate TIME for scientists to further explore the interactions between PCa cells and immune system under in vitro and in vivo conditions.

Extensive studies have been performed to investigate the cross-talk between tumor and immune cells in 2D culture models. These studies have shed light on specific immune cell types either in conditioned media obtained from PCa or directly co-culturing immune cells with PCa cells. To develop these 2D models in the context of TIME, an immortalized lymphocyte line (Jurkat cells, or primary immune cells collected from PBMCs) was co-cultured with PCa cell lines [83]. However, these models were shown to have limited effects on tumor/immune interactions for immunotherapeutic applications. Recently, Lee et al. developed engineered chimeric antigen receptor T (CAR-T) from PBMC-derived T cells for targeting CEACAM5 (a protein upregulated in tumor cells) to provoke cytotoxicity in neuroendocrine PCa (NEPC) cell lines [84].

Several PCa models have been established to explore PCa biology in the context of TIME in the past years. In 2022, Yamaguchi and his research team established an in vitro system by co-culturing PCa cell lines with CAR-T cells and PBMC derived macrophages [85]. The study revealed that M2 macrophages enhanced CAR-T activity against PCa cells under the in vitro condition, indicating the clinical significance of including multiple immune compartments for testing immunotherapeutic effects in vitro. However, these strategies were only favorable at an early stage for developing novel immunomodulatory approaches, and PCa heterogenous TIME need to be established in an improved 3D model for evaluating the clinical significance of novel immunotherapies for PCa patients.

In terms of 3D tumor models for immunotherapy studies, TIME should be considered when a variety of 3D tumor models are established. These 3D models include the 3D cultures from PDOs, PDEs and microfluidic systems [86,87]. To more effectively elucidate the cross-talk between immune cells and non-immune compartments of cancer cells, it is critical to ensure that the immunotherapy study is conducted in a tissue-derived organoid model or a 3D co-culture system combining cancer and immune cells. PDOs and explant cultures have been demonstrated with an advantage of sustainable host-resident immune cells; however, the poor viabilities of such 3D cultures hinder their significance for immunomodulatory studies. To address this, establishment of an optimal culture that maintains epithelial/stromal components with immune cell population is in need for future immunotherapeutic studies in PCa. To establish a workable in vitro 3D culture similar to PDO models, co-culture systems containing PBMC-derived cells (or immune cells isolated from tissues) and tumor organoids have been developed. These in vitro models, with optimization of culture conditions with immune population of interest, have been utilized to explore the molecular mechanisms underlying immunotherapy in PCa [88,89].

Although an organoid system with complex PCa-TIME interaction remains to be developed, recent studies have highlighted the promising progress in the development of tumor–immune interaction systems in other solid tumors. For instance, immune profiling studies on non-small cell lung cancer (NSCLC), renal cancer, and melanoma PDOs with TIME have revealed that the CD8^+^ and CD4^+^ T cells, CD14/CD68/CD 69^+^ macrophages, NK (natural killer), NKT (natural killer T) and B cells were well-maintained in tumor PDO models [7,90,91]. These results suggested that PDO models can be efficiently utilized for testing novel immunotherapeutic strategies for provoking/activation of T cells. Particularly, these models can be used to evaluate the efficacies of immune checkpoint blockers (such as anti-PD-1 antibody) on activating T cell to against PCa cells. Furthermore, it has been reported that human pancreatic organoids could be effectively co-cultured with cancer-associated fibroblast and human peripheral T cells, successfully demonstrating T cell infiltration in a PDO model [86,92,93]. Additionally, previous studies have shown that co-culturing NSCLC or colon organoids with matched PBMC cells resulted in an increase in the CD8^+^ anti-tumor T cell population [94]. Notably, it has been reported that a metabolic shift in tissue microenvironment contributes to the recruitment and activation of immune cells, particularly when pembrolizumab (anti-PD-1 antibody) was applied to NSCLC model containing TIME [95,96].

In summary, development of PCa organoid systems considering prostate TIME holds promise for scientists to further develop personalized immunotherapeutic strategies for PCa patients.

#### 3.1.7. In Vivo Implantation of PCa Organoids

In recent years, several studies have been conducted by implanting organoids in mouse models [97,98,99]. Park et al. [99] developed a PCa organoid model using oncogene-transduced human primary prostate basal and luminal cells. Their study suggested that the luminal cells are involved in later stages of oncogenic transformation during the development of PCa. These oncogene-transformed organoids could further be developed into atypical and/or glandular architecture once these organoids were used to develop xenografts in the immunodeficient NOD-SCID mice [100]. Specifically, PCa organoids, derived from basal or luminal organoids, were collected and subcutaneously implanted into immunodeficient mice. The xenografts derived from c-Myc/AKT-transduced basal organoids exhibited histological features of poorly differentiated adenocarcinoma, while the xenografts derived from luminal organoids displayed characteristics of low-grade adenocarcinoma.

A previous study also showed that PCa organoids were orthotopically implanted into C57BL/6J mice for investigating intra-epithelial Activin A signaling, a signaling pathway critical for progenitor proliferative potential in response to the microenvironment in prostate epithelium. In the mouse model implemented with prostate organoids, it was shown that the intra-epithelial Activin A signaling inhibits cell proliferation, independent from regulation by Smad. This finding implicates a critical role of Activin A signaling in healthy prostate cells, potentially paving an alternative avenue for developing novel therapies by targeting quiescent tumor progenitors [101].

The strengths and limitations of PCa organoid models mentioned in Section 3 are summarized in Table 1.

## 4. Clinical Implications and Therapeutic Approach

PCa organoids have also been used to characterize PCa subtypes and screen for effective drugs [21,36,108,109,110]. Twenty organoids generated from the LuCaP mCRPC PDX cohort (PCa PDX cohort with distinct LuCaP features, such as adenocarcinoma and neuroendocrine phenotypes) were established. Among these PDX-derived organoids, an organoid system derived from treatment-naïve metastatic PCa was particularly selected for the drug screening procedure [14]. It has been reported that mutations on the DNA damage repair (DDR) genes, such as *BRCA1/2*, *ATM*, *CDK12*, *RAD51C* and *FANKA*, are frequently detected in primary PCa and mCRPC [111]. Moreover, the mutations/variations in DDR genes also caused differential responses of PCa to PARP inhibitors (inhibitors that cause synthetic lethality in DDR-mutated cancers). For example, PARP inhibitors have been used to treat cancers carrying *BRCA1/2* mutations [112,113,114]. PCa organoids (such as PCa PDOs) were utilized to serve as 3D models for assessing the drug efficacies of targeted therapies against aggressive PCa [115]. Specifically, PCa organoids derived from NEPC patients were developed and employed as novel models to study pathogenesis of NEPC, leading to the identification of ALK as a promising drug target [116,117].

On the other hand, PCa organoids derived from mCRPC patient biopsies were developed and selected for studying efficacies of targeted therapeutic drugs, such as BET domain inhibitors [118]. For example, one of the studies suggested that the combination of CX-5461 (RNA polymerase I inhibitor) and CX-6258 (pan-PIM kinase inhibitor) exerts potent anti-tumor activities against the PCa organoids derived from PDXs of advanced PCa [119].

A high-throughput screening (HTS) approach has been demonstrated as a powerful method for screening effective cancer drugs. Previously, the scientists established an efficient HTS platform for testing novel anti-tumor compounds/drugs by including 100 micro-tumors in 48-well plates [120]. The success rate for developing PDO models is a key for establishing an efficient HTS platform of cancer drugs in clinical trials. Novel tumor organoids developed from distinct cancer subtypes (based on immunohistochemical classification) have been considered as promising 3D models for screening/evaluation of novel cancer drugs. Herein, we summarized recent clinical studies that utilized PCa organoids and model systems for evaluating the novel therapies for PCa diseases (Table 2). These studies included the clinical trials conducted at Centre Antoine Lacassagne in France (NCT03952793) [121], The Christie NHS Foundation Trust in the UK (NCT04723316) [122], the Netherlands Cancer Institute in the Netherlands (NCT02695459), Fudan University in China (NCT04927611) [123], SpeciCare, Georgia, USA (NCT03896958) [124], and the Rutgers Cancer Institute in New Jersey (NCT02458716) [36] (Table 2). Recently, a PDO-based model was employed in a Phase II trial (NCT01799278) for investigating the efficacy of the aurora kinase inhibitor Alisertib (MLN8237) for mCRPC and NEPC. Although this clinical study did not meet its primary endpoint, a significant response to Alisertib was observed in a subset of NEPC PDOs due to aurora/N-Myc targeting [26].

## 5. Limitations in Establishment of PCa Organoids

Despite the novelty of PCa organoid development, several limitations of PCa organoids were noted. First, the overall success rate of PCa organoids is low, ranging between 15 and 20% [17]. Thus, there is an urgent need to improve the success rate of developing an organoid (i.e., by optimizing the culture conditions). In addition, development of organoids from primary PCa patients has been reported with partial/limited success [125,126]. Moreover, it has also been reported that the major components of PCa organoid are epithelial and stromal cells, and a lower percentage of tumor cells has been detected. The scarcity of accessibility of clinical PCa and mCRPC specimens also hinders the development of a PCa organoid bank with a broad range of PCa subpopulations [18]. Taken together, a more feasible PCa organoid model for PCa study is yet to be developed/optimized. Further development of PCa organoid models from PCa specimens with different clinical features (i.e., primary/metastatic PCa, mCRPC, and NEPC) will facilitate the application of PCa organoids in translational oncology research.

## 6. Application of PCa Organoid Development

**Figure 3:**
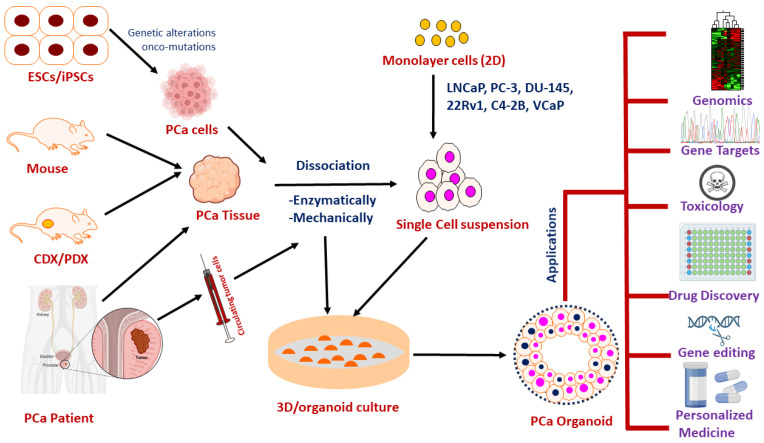
The summarized picture of a differential PCa culture from embryonic stem cells (ESCs) and induced pluripotent stem cells (iPSCs) transformed after the carcinogenesis process, mouse PCa, cell line-derived xenograft (CDX) or patient-derived xenograft (PDX) from mice, and tissue or circulating tumor cells of a PCa patient. PCa cells and/or tissues are also developed for a monolayer (2D) cell culture model and are subject to be developed to 3D organoid cultures. PCa representative organoids can be utilized for different applications including genomics, toxicology, gene editing, drug development and personalized medicine.

As a promising/novel technology, tumor organoids have been implicated as a crucial tool for basic, preclinical, and clinical research. Several investigations have demonstrated that PCa organoids are not only recapitulating in situ histological characteristics/alterations, but also serving as model in vitro systems for investigating alterations in genomic landscapes during PCa development and/or progression [17,102,105,127]. Moreover, PCa organoids are ideal platforms for investigating drug efficacies against PCa diseases. The broad applications of PCa organoid models are summarized in Figure 3 and described in the following sections.

### 6.1. Development of PCa Organoids for Revealing Gene Regulatory Networks

The cellular machinery driving oncogenesis of PCa is yet to be elucidated, and the subgrouping/subtypes of PCa based on pathological/histological states remain to be further characterized. The genome editing technology, as mentioned above, can be potentially applied to establishing PCa organoid models for a functional study of molecular mechanisms underlying PCa diseases [55,128]. Among various in vitro models, an efficient PCa organoid system potentially opens a new avenue for the mechanistic studies of PCa diseases. For instance, CRISPR/Cas9-based genome editing and ectopic expression of oncogene/tumor suppressor gene (by lentiviral or plasmid transfection) can be employed to explore the molecular mechanisms underlying PCa development/progression [129,130,131].

Chua et al. [19] established PCa organoid cultures from castration-resistant Nkx3.1-expressing cells (CARNs) containing luminal and basal cells. By deleting *PTEN* and activating KRASG12D through genetic manipulations, the AR signaling pathway is upregulated in the PCa organoids derived from castration-resistant Nkx3.1-expressing cells. This finding highlighted the critical roles of *PTEN* deletion and activated *KRAS* mutation in the development and progression of PCa.

PCa organoids have also been employed to highlight TIP5-mediated PTEN downregulation. It has been shown that loss of TIP5 resulted in downregulation of PTEN and activation of oncogenic transformation in prostate luminal cells [132,133]. These studies have suggested that TIP5-mediated chromatin remodeling may play a critical functional role in PTEN-mediated tumor suppressive signaling.

Due to tumor heterogeneity, it is challenging to identify the driver mutations in PCa diseases. Therefore, organoid models with PCa heterogeneity may provide a unique opportunity that benefits the PCa research community [134,135,136]. The normal and cancer tissue-derived organoids obtained from the same patient could be used to compare gene expression profiles and identify differential activation of specific oncogenic signaling pathways in PCa. Furthermore, a normal tissue-derived organoid is comparatively more stable in genetic information and may be utilized as a model for evaluating the functional roles of specific tumor mutations for driving PCa oncogenesis [132].

### 6.2. Establishment of PCa Organoids in Drug Screening

PCa organoids retain genetic features and tumor heterogeneity, serving as ideal 3D models for HTS of anti-cancer drugs through examining drug efficacies and toxicities [137]. For example, CRPC-derived organoids have been used to evaluate the efficacies of enzalutamide (an AR antagonist) [25,103,138], everolimus (a mTOR inhibitor) [72,139], and BKM-120 (a PI3K inhibitor) [17,140].

Furthermore, it has been shown that the MSK-PCa2 (Memorial Sloan Kettering-Prostate Cancer-2) organoid was significantly sensitive to enzalutamide. However, the other organoids (derived from PCa cell lines) were resistant to enzalutamide. These results were similar to the findings in previous clinical trials. Compared to wild-type MSK-PCa2 organoid, MSK-PCa2 organoid with *PTEN* deletion and *PIK3R1* mutation have shown to be more sensitive to everolimus and BKM-120. These results were consistent with the previous in vivo studies [17]. Overall, these results suggested that organoids can be utilized as excellent platforms for mechanistic and drug screening studies, potentially facilitating the development of individualized treatments.

Previous studies further showed that 4 CRPC-neuroendocrine (CRPC-NE) organoids and 2 CRPC-adenocarcinoma (CRPC-Aden) organoids were established for HTS of 129 chemo and targeted therapeutic drugs. The results suggested higher efficacies of an AR antagonist (such as enzalutamide) and other chemotherapeutics (such as cabazitaxel and docetaxel) in CRPC-derived organoids [22,105]. Drug candidates, such as pozotinib and vandetanib, were also tested in PCa organoids. Both drugs have been demonstrated to possess significantly inhibitory capacities against CRPC-NE organoids [22]. Previously, Jansson and colleagues performed an HTS for >100 drugs in CRPC-LuCaP PDX-derived organoids. Among the drugs, heat shock protein-90 (HSP90) inhibitors were shown with significant anti-tumor activities in CRPC-LuCaP PDX-derived organoids [141]. One of the HSP90 inhibitors, ganetespib, particularly exhibited the strongest inhibitory effect against CRPC-LuCaP PDX models via targeting AR and PI3K signaling pathways [141].

Recently, our research group evaluated the efficacies of the Idelalisib, SRPIN340, and Idelalisib/SRPIN340 combination in 3D spheroids derived from PCa and other solid tumor cell lines (22Rv1, PC-3, LNCaP, MDA PCa 2b, DU-145, C4-2B, HT-29, SW620, A549, H1299, MSA MB 231, and MCF-7). Our results suggested that combined Idelalisib/SRPIN340 therapy resulted in a synergistic effect on inhibiting PCa and other solid tumor spheroids expressing PI3Kδ isoforms [142]. Additionally, we established PCa organoids from LNCaP, C4-2B, 22Rv1 and MDA PCa 2b cells for exploring the mTOR/AR signaling in the in African American (AA) PCa disparities and CRPC progression. The results suggested that downregulation of tumor suppressive miR-99b-5p mediates the upregulation of the mTOR/AR/SMARCD1 signaling axis, consequently promoting tumor aggressiveness and metabolic reprogramming in AA PCa and CRPC [143]. These two studies highlighted the importance of PCa spheroids and organoids as efficient 3D models for evaluating drug effects and molecular mechanisms underlying PCa.

### 6.3. PCa Organoid Models for Exploring Drug Resistance Mechanisms

Gene mutation, chromosomal amplification and rearrangement are the major causes for tumor cells to develop drug resistance [144,145,146]. PCa organoids can also serve as in vitro or ex vivo 3D models for exploring the molecular mechanisms underlying drug resistance [147]. To date, three major mechanisms have been proposed for the development of drug resistance in CRPC: (1) mutations in *AR*, *ETS*, *TP53*, and *PTEN* genes, alterations in AR signaling, and alterations in the PI3K signaling pathway [148]; (2) aberrant RNA splicing, which results in a signaling shift from AR to glucocorticoid receptor signaling [149]; (3) acquired resistance, which is developed due to prompted lineage plasticity after prolonged drug treatment that results in AR-negative or neuroendocrine PCa [150,151].

PCa organoids were also used as model systems for elucidating the molecular mechanism in the mutation-derived drug resistance. Pappas and colleagues utilized the PCa organoid as a model system to study the efficacies of anti-androgen drugs against p53 and PTEN-deficient PCa [152]. In addition, several studies highlighted the roles of *SPOP* mutations in promoting the resistance of BET (bromodomain and extra-terminal) inhibitors [57,153,154]. In addition, drug-resistant organoids can be developed directly from PCa patients with specific resistance to anti-cancer drugs or developed in vitro by inducing the resistance through long-term exposure to the anticancer drugs in the organoid model [136]. For example, PCa organoids resistant to ADT have been developed by culturing PCa cells in the absence of androgen or in the presence of the anti-androgen agent, such as enzalutamide [155]. In 2021, Dhimolea and the team [156] investigated treatment-resistant tumor cells in organoids, xenografts, and cancer patients. Their study showed that suppression of BRD4, an MYC co-activator, resulted in reduced drug cytotoxicity and promoted drug resistance. Also, organoid cultures have been utilized in studies for evaluating the relationship between gene mutations (i.e., via CRISPR/Cas9 editing) and drug resistance [55,157]. Recently, a synthetic hydrogel-based organoid model with putative extracellular matrix (ECM) was employed to examine the drug effects of EZH2 and DRD2 inhibitors in CRPC-NEPC. Proteomics, RNA sequencing, special-omics and functional assay results have suggested that epigenetic inhibitors followed by DRD2 treatment can overcome the drug-resistant ECM conditions observed in CRPC-NEPCs [158].

Taken together, PCa organoids are considered as promising model systems for systematically evaluating the efficacies of therapeutic drugs and investigating the molecular mechanisms underlying drug resistance in PCa [159,160].

### 6.4. Possible Role of PCa Organoids in Personalized/Precision Medicine

Several studies have highlighted the opportunity for applying PCa organoids in precision medicine. PCa organoids have been suggested as promising in vitro or ex vivo models to study the drug efficacies/responses, and the results can serve as good references for predicting clinical outcomes [14,26,36,161,162]. In fact, PDO models have been used to evaluate drug efficacies in several clinical trials for PCa diseases. For instance, Puca and co-workers conducted a clinical trial for HTS of drugs in PCa organoid models derived from CRPC-NE patient specimens. In this study, one CRPC-NE organoid was shown with a marked sensitivity to an aurora kinase inhibitor, alisertib [22]. This observation was consistent with the drug responses in a clinical trial (NCT01799278) in which alisertib exhibited significant effects in a subgroup of CEPC-NE patients [26,105].

In addition, Beshiri et al. [21] showed that LuCaP-derived organoids with deficient *BRCA2* were sensitive to olaparib in a clinical trial [163]. A few preclinical models derived from CRPC were utilized as in vivo systems for testing the drug efficacies of combination of the PARP inhibitor (such as olaparib) and cisplatin [164]. In 2023, Zhang and his team showed that Z15 (an AR antagonist and selective AR degrader) can effectively block/degrade AR/AR-V7 activity/level in CRPC cell lines and organoids [165]. These studies indicate that organoids may serve as a powerful tool in clinical studies for developing novel therapeutic strategies for PCa. More recently, a series of PDX models were developed from different stages of PCa, including hormone-naïve PCa, androgen-sensitive PCa, CRPC and CRPC-NE [166]. These PDX models represent the complexity of PCa heterogeneity and potentially serve as ideal resource for scientists to further develop PDX-derived organoids for precision medicine of PCa diseases at different stages.

Taken together, PCa organoids may serve as powerful tools for evaluating drug efficacies/responses in PCa clinical trials and/or effective models for investigating molecular mechanisms underlying PCa diseases.

## 7. Conclusions and Future Perspectives

The development and availability of PCa organoids provide a unique opportunity for the scientific community to utilize these 3D models as novel platforms to elucidate molecular mechanisms associated with PCa development/progression and to evaluate the efficacies of new anti-tumor drugs. Compared to 2D cultures, 3D organoids derived from PCa tissues serve as more sophisticated models that recapitulate the critical biological properties of PCa diseases. In addition, PCa tissue-derived organoids reserve the genomic makeup and molecular/genetic alterations of the tumors from PCa with different clinical characteristics.

Despite the many advantages of 3D organoids in PCa research, several challenges and limitations remain to be resolved. One of the challenges is the low success rate for establishment of patient-derived PCa organoids. Compared to the high success rate of organoids derived from other cancers (for example, 90% success rate for establishing colorectal cancer organoids [167]), the success rate remains as low as ~20% for developing PCa organoids. The low success rates of establishing PCa organoids may reflect the different sources and intrinsic characteristics of PCa patient samples used for developing organoids. To further improve the culture condition for PCa specimens from different sources. it may help to obtain a higher success rate for developing novel PCa organoid models.

Another challenge is to establish PCa organoids from a series of patient specimens with different clinical characteristics, such as primary PCa, metastatic PCa, mCRPC, and NEPC. It is indeed labor intensive and costly for coordinating clinicians with researchers to collect fresh patient samples. An efficient procedure needs to be implemented for patient sample collection, ultimately developing a “biobank” containing a broad range of PCa populations (i.e., from cell lines and patient samples with distinct clinicopathological features). These PCa organoid models from the “biobank” can then serve as models for multiple downstream investigations, including studying molecular mechanisms, validating potential diagnostic/prognostic biomarkers, and developing novel therapeutics in PCa diseases. One example is the application of organoid models in CRPC research. It is known that the high treatment selection pressure in CRPC patients significantly increases PCa heterogeneity. It would be critical to develop organoid models that reflect the entire process of PCa transformation. Such organoid models will allow scientists to investigate the molecular events/alterations during the process of developing CRPC, NEPC, and metastasis.

In addition, PCa has been considered as an “immunologically cold” cancer in previous studies. The current PCa organoids containing epithelial cells and/or stromal cells are not ideal models for immunotherapy studies. PCa organoid models co-cultured with peripheral blood lymphocytes may serve as more efficient models for testing the cytotoxic effects of T cells in response to novel immunotherapeutic drugs. Moreover, combination of PCa cells with stromal, immune cells, and/or vascular components will further facilitate the establishment of PCa organoid systems equipped with immune compatible microenvironment. These novel PCa organoids with the context of TIME will be utilized as novel models for future immunotherapy studies.

In conclusion, PCa organoids are considered as novel/promising in vitro preclinical models for broad applications. These models demonstrate genetic stability and heterogeneity of PCa diseases and can be applied to in vitro functional and mechanistic studies. For instance, PCa organoids can be offered as effective models for studying the molecular mechanisms underlying ADT resistance and to screen for potential therapeutics. PCa organoids could further serve as platforms for HTS of anti-tumor drugs, facilitating the development of novel therapeutic regimens for personalized medicine. The improvement of PCa organoid models with combination of immune compatible microenvironment will further pave a new path for developing novel immunotherapeutic strategies against PCa diseases.

## Figures and Tables

**Table 1:** Strengths and Limitations of current PCa organoid models.

Model	Strength	Limitations	References
iPSC-derived organoid	Enables genome characterization; High-throughput drug screening; Retains heterogeneity; Different source of iPSC; Ability to recapitulate in vivo tissues.	No observation of immune influence; Lack of tumor microenvironment	[102,103]
Cell line-derived organoids	An indefinite expansion of cells can be considered; Easy and cost-effective to grow; Enables genome characterization; High-throughput drug screening; Retains heterogeneity; Ability to recapitulate in vivo tissues.	Limited to 2D culture; Probability of mutations over time; Lack of immune-mediated tumor microenvironment.	[25,104,105]
Patient-derived Organoids (PDOs)	Enables genome characterization; High-throughput drug screening; Retains 3D heterogeneity or tissue architecture; Resemblance to tissue of origin at molecular and histological level.	No observation of immune influence; Low efficiencies of tumor organoid establishment from prostatectomy; Up to now, only aggressive PCa are established as organoid lines.	[36,106]
Patient-derived xenograft (PDX)-formed organoids	Enables genomic characterization; Organoids can be applied for PDX grafting for long-term functional investigations.; Retains tumor heterogeneity; Ability to recapitulate in vivo tissues; High-throughput drug screening.	Low success rates for developing CRPC organoids with long-term/stable phenotypes; Prolongs time for establishment; Expensive; Low engraftment efficiencies; Mouse has deficient immunity and different microenvironment.	[25,102,107]

**Table 2:** The PCa organoids used in clinical trials.

No.	Trial ID	Sponsor/Location	Origin	Measure Outcomes	Enrollment
1.	NCT03952793	Centre Antoine Lacassagne, Nice, France	Metastatic biopsies	Prostatic neoplasm: Development of the organoid technique from metastases of patients with an advanced form of prostate cancer.	20
2.	NCT04723316	The Christie NHS Foundation Trust, the United Kingdom	Blood Specimens	Establishment of a framework to offer molecular profiling of circulating tumor DNA and/or tumor tissue to patients with advanced cancers.	6000
3.	NCT02695459	The Netherlands Cancer Institute, Netherland	Metastatic neuroendocrine carcinoma patient’s blood and tissue biopsies	Tumor biopsy included for DNA/RNA analyses and organoid culture; from cisplatinum and everolimus treatment, the experimental patients showed considerable response with high success rate.	39
4.	NCT04927611	Fudan University, Shanghai, China	Biopsies/surgical fresh tissue from neuroendocrine neoplasm	Analysis of neuroendocrine molecular biology information by using single cell sequencing technology; exploration and optimization of the medium strategies of the neuroendocrine organoid and methods of histomorphology identification in tumor microenvironment.	200
5.	NCT03896958	SpeciCare, Georgia, United States	Biospecimen tumor tissues from all types of cancers including PCa	Functional precision testing of a patient’s tumor tissue in organoid-mediated drug screening via traditional genomic profiling.	200
6.	NCT02458716	Rutgers, The State University of New Jersey	Biopsies of metastatic prostate cancer	Prostate-specific antigen (PSA) and castration resistance following cytoreductive prostatectomy and subsequent standard systemic therapy, androgen deprivation.	26
7.	NCT04497844	Janssen Research and Development, LLC, United States	Radiographic progression in a solid tumor of prostate cancer.	A study of Niraparib in combination with Abiraterone Acetate and Prednisone versus Abiraterone Acetate and Prednisone for the Treatment of Participants with deleterious germline or somatic homologous Recombination Repair (HRR) Gene-Mutated Metastatic Castration-Sensitive Prostate Cancer (mCSPC).	696
